# Metal Binding to Sodium Heparin Monitored by Quadrupolar NMR

**DOI:** 10.3390/ijms232113185

**Published:** 2022-10-29

**Authors:** Daniel Sieme, Christian Griesinger, Nasrollah Rezaei-Ghaleh

**Affiliations:** 1 Department of NMR-Based Structural Biology, Max Planck Institute for Multidisciplinary Sciences, Am Faßberg 11, D-37077 Göttingen, Germany; 2 Institute of Physical Biology, Heinrich Heine University (HHU) Düsseldorf, Universitätsstrasse 1, D-40225 Düsseldorf, Germany; 3 Institute of Biological Information Processing, IBI-7: Structural Biochemistry, Forschungszentrum Jülich, Wilhelm-Johnen-Straße, D-52428 Jülich, Germany

**Keywords:** metal, sodium, heparin, NMR, diffusion, relaxation, quadrupolar nuclei

## Abstract

Heparins and heparan sulfate polysaccharides are negatively charged glycosaminoglycans and play important roles in cell-to-matrix and cell-to-cell signaling processes. Metal ion binding to heparins alters the conformation of heparins and influences their function. Various experimental techniques have been used to investigate metal ion-heparin interactions, frequently with inconsistent results. Exploiting the quadrupolar ^23^Na nucleus, we herein develop a ^23^Na NMR-based competition assay and monitor the binding of divalent Ca^2+^ and Mg^2+^ and trivalent Al^3+^ metal ions to sodium heparin and the consequent release of sodium ions from heparin. The ^23^Na spin relaxation rates and translational diffusion coefficients are utilized to quantify the metal ion-induced release of sodium ions from heparin. In the case of the Al^3+^ ion, the complementary approach of ^27^Al quadrupolar NMR is employed as a direct probe of ion binding to heparin. Our NMR results demonstrate at least two metal ion-binding sites with different affinities on heparin, potentially undergoing dynamic exchange. For the site with lower metal ion binding affinity, the order of Ca^2+^ > Mg^2+^ > Al^3+^ is obtained, in which even the weakly binding Al^3+^ ion is capable of displacing sodium ions from heparin. Overall, the multinuclear quadrupolar NMR approach employed here can monitor and quantify metal ion binding to heparin and capture different modes of metal ion-heparin binding.

## 1. Introduction

**Scheme 1:**
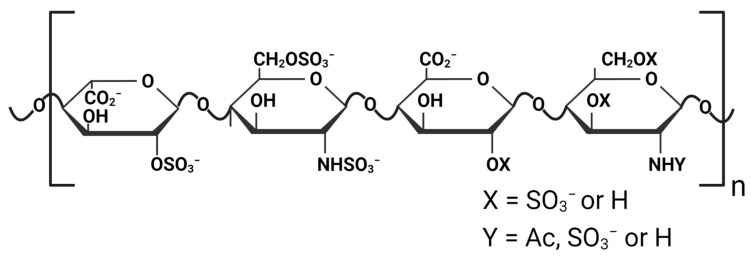
Chemical structure of heparin is shown as an example by two disaccharide units: the left disaccharide unit composed of 2-O-sulfated iduronic acid and 2-N- and 6-O-sulfated glucosamine, and the right disaccharide unit composed of glucuronic acid (with or without 2-O-sulfation) and glucosamine (with or without 2-N-acetylation or sulfation or 6-O-sulfation). Created with Biorender.com.

Heparins and heparan sulfate polysaccharides are polydisperse linear glycosaminoglycans (GAGs) composed of repeating disaccharide units of D-glucosamine and uronic acid residues. The O- and N-sulfation of these disaccharide units lead to the high negative charge of these GAGs (Scheme 1) [1]. Heparins are commonly found in the granules of mast cells, and heparan sulfates are ubiquitously present in the extracellular matrix of a wide variety of animal tissues and organs [2,3]. These polyanionic GAGs interact with a diverse array of biomolecular and biological targets, including extracellular matrix and cell surface components, growth factors, proteases, protease inhibitors, and various pathogens [4,5,6]. They play significant roles in many pathophysiological processes, such as cell-to-cell and cell-to-matrix signaling, inflammation, cellular growth and differentiation, angiogenesis, blood coagulation, and host defense against pathogens [5,7,8]. In recent years, the pharmacological application of heparin and heparin-like GAGs has extended from their classic use as anti-coagulant and anti-thrombotic agents to potential anti-inflammation, neuroprotective, antiviral, and anti-cancer therapeutics [1,9,10].

Due to the high concentration of negatively charged sulfate and carboxylate groups, heparin molecules exhibit high binding affinity to positively charged metal ions. It has been shown that heparin binds to monovalent cations such as Na^+^ and K^+^, divalent cations such as Ca^2+^ and Mg^2+^, and trivalent cations such as Al^3+^, among others [11]. Heparins can serve as a reservoir of metal ions in the extracellular environment and regulate metal ion-dependent biomolecular activities [12]. In addition, the binding of heparin to metal ions can alter the three-dimensional structure of heparin, hence modulating its interaction with various biomolecular targets [10,12,13].

The binding of metal ions to heparin has been previously studied through techniques such as infrared spectroscopy, atomic absorption, circular dichroism, optical polarimetry, NMR spectroscopy, and potentiometric titration [11,14,15,16]. These techniques require different sample conditions and are sensitive to various aspects of metal ion-heparin binding; hence the results have frequently been inconsistent. Here, we employ NMR spectroscopy, specifically ^23^Na NMR relaxation and diffusion methods, to investigate metal ion binding to sodium heparin through competition-based assays. The ^23^Na NMR has been used to study sodium ion binding to various biological macromolecules, including heparin [16,17]. More recently, it has been demonstrated that ^23^Na NMR, along with ^35^Cl and ^17^O NMR, can serve as a sensitive proxy to monitor ions and water dynamics in highly concentrated and crowded aqueous solutions and confined environments [18,19,20,21,22]. ^23^Na has a natural abundance of about 100% and a gyromagnetic ratio of 11.262 MHz.T^−1^, which is around 0.26 of ^1^H nuclei [23]. As a result, ^23^Na is a fairly sensitive nucleus in NMR experiments. It has a spin quantum number of 3/2 with a quadrupole moment (Q) of +0.104 Barn [24], which indicates a rather strong quadrupolar relaxation provided that there is a sizeable electric field gradient at the site of ^23^Na nuclei. Consequently, the ^23^Na NMR signals of sodium-containing complexes are often severely broadened due to the large electric field gradient and the resultant efficient quadrupolar relaxation [25,26]. This is the case for sodium heparin where the positively charged ^23^Na ions are bound through ionic interaction with the negatively charged sulfate and carboxylate groups. On the other hand, the hydrated sodium ions in aqueous solutions experience a nearly symmetrical electric environment with a near-zero electric field gradient and therefore possess a low quadrupolar relaxation rate and can lead to relatively narrow ^23^Na NMR signals [18,23]. Exploiting the difference in NMR behavior of heparin-bound sodium and free hydrated sodium ions, we determine the relative heparin-binding affinity of divalent cations Ca^2+^ and Mg^2+^, as well as the trivalent cation Al^3+^. In the latter case, we combine ^23^Na NMR with ^27^Al NMR measurements and simultaneously monitor the binding of Al^3+^ ions to heparin and the resultant release of Na^+^ ions from heparin. Our results demonstrate the presence of more than one metal ion-binding site on heparin and determine the order of binding affinity for the studied ions. More generally, this study highlights multinuclear quadrupolar NMR’s potential in quantifying metal ion binding to heparins and distinguishing various modes of binding.

## 2. Results

To begin with, a brief overview of the theory of ^23^Na NMR relaxation in solution is presented. As a quadrupolar nucleus, the NMR relaxation of spin-3/2 ^23^Na is dominated by a quadrupolar relaxation mechanism. Energy fluctuations in the system cause quadrupolar NMR relaxation due to the anisotropic interaction between the electric quadrupole moment of the nucleus (*eQ*) and the electric field gradient (EFG) tensor present at the site of nuclei. The rate of quadrupolar relaxation is determined by the magnitude and timescale of fluctuations in the energy of this anisotropic interaction, mainly caused by stochastic rotational motions of molecules in solution and their associated EFG tensors. According to Redfield’s theory, the quadrupolar relaxation of a spin-3/2 nucleus is generally governed by a bi-exponential relaxation process where the low and high relaxation rates correspond respectively to the central transition (|−1/2〉⟷|+1/2〉) and one pair of satellite transitions (| ± 1/2〉⟷| ± 3/2〉). However, if the rotational correlation time, τ_c_, is much smaller than 1/ω_0_ (ω_0_, Larmor frequency of ^23^Na in rad. s^−1^, 1.5 ns in our study), the two relaxation rates of ^23^Na nuclei become identical, and a mono-exponential relaxation process is recovered. Consequently, under this so-called fast “extreme-narrowing” regime, the ^23^Na signal line shape becomes single-Lorentzian, and the ^23^Na longitudinal spin-lattice and transverse spin-spin relaxation times, *T*_1_ and *T*_2_, respectively, are identical, independent of ω_0_ (as long as it remains within the extreme-narrowing regime), and inversely proportional to τ_c_. The ^23^Na relaxation times can therefore represent the rotational mobility of sodium ions in this regime. On the other hand, in the “slow motion” regime where $\omega_{0}\tau_{c}\;\sim \;1$ or larger, the central and satellite transitions will experience distinct *T*_1_ and *T*_2_ relaxation times, which are field-dependent through dependence on spectral densities *J*(ω_0_) and *J*(2ω_0_). Due to different *T*_2_ relaxation times for the two central and satellite transitions, the ^23^Na signal line shape becomes double-Lorentzian. When $\omega_{0}\tau_{c}$ increases due to an increase in ω_0_ and/or τ_c_, the two *T*_1_ relaxation times decrease until they reach their minimum values and then monotonously increase afterward. The *T*_2_ relaxation time of the central transition follows a similar trend; however, the satellite transition exhibits a monotonous decrease with $\omega_{0}\tau_{c}$ [17,25,26,27]. With further increase in τ_c_ when $\omega_{0}\tau_{c}>{\left(\frac{\omega_{0}}{\omega_{Q}}\right)}^{2}$ (ω_Q_ is the quadrupolar coupling constant in angular frequency units), the system enters the so-called “ultraslow regime,” and its relaxation deviates from Redfield’s theory, which is outside the scope of the current study (see Methods for further description) [26,28].

### 2.1. Titration with Metal Ions Leads to Sodium Ion Release from Heparin

**Figure 1:**
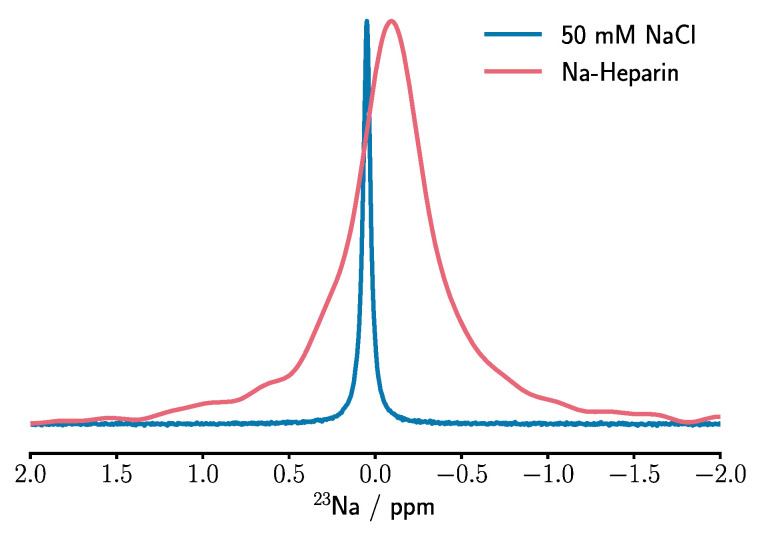
1D ^23^Na NMR spectrum of the sodium heparin sample (red), showing a significant signal broadening and a small displacement towards negative chemical shifts when compared to the reference NaCl sample (blue).

First, we measured the 1D ^23^Na NMR spectrum of a control sodium chloride solution in water (50 mM), which resulted in a single peak with an (apparent) chemical shift of 0.05 ppm (Figure 1). The peak was well fit by a single Lorentzian, and the linewidth obtained through the fit was ca. 5.3 ± 0.1 Hz. Then, the 1D ^23^Na NMR spectrum of an aqueous solution of sodium heparin (5 IU/mL) showed a single rather broad peak with an apparent linewidth of 48.3 ± 0.5 Hz at around −0.095 ppm, which was again well fit by a single-Lorentzian. As mentioned above, the almost ten-fold increase in the linewidth of the ^23^Na NMR signal is expected to have been caused by a combination of longer rotational correlation time and larger local EFG present in the place of heparin-bound sodium ions when compared to the free symmetrically hydrated sodium ions in the control solution. The slight upfield chemical shift change is likely to represent the relative shielding of ^23^Na nuclei in the heparin-bound form because of the nearby negative charges. The comparison with integrated signal intensities of known sodium chloride solutions (10 mM, 50 mM, and 1 M) indicated that around 44 ± 2 mM of sodium ions contributed to the signal in the measured sodium heparin samples.

**Figure 2:**
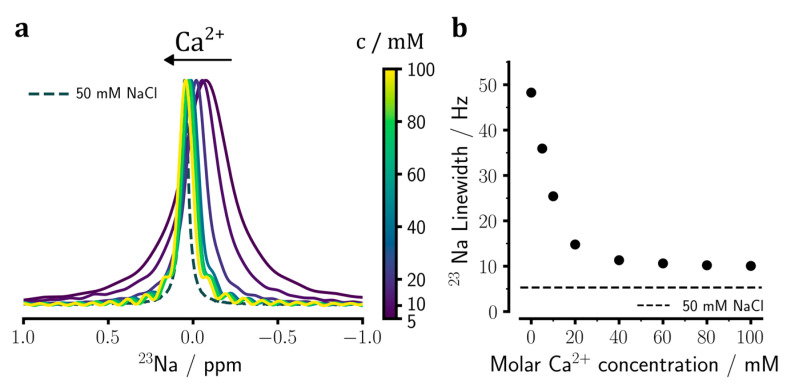
^23^Na NMR spectral changes of sodium heparin caused by the addition of Ca^2+^ ions and the consequent release of sodium ions from heparin. (**a**) Upon titration of sodium heparin with 0 to 100 mM CaCl_2_, the ^23^Na chemical shift gets narrower and moves downfield towards the chemical shift of the reference NaCl sample (see Figure 1). (**b**) Changes in the ^23^Na signal linewidth in dependence of added Ca^2+^ ion concentration, showing a monotonic decrease approaching a value slightly larger than the linewidth of the reference NaCl sample (represented by the dashed line). The spectra shown in (**a**) are re-scaled according to their peak intensities. The error bars in (**b**) are comparable in size to the symbols and, therefore, barely visible.

Next, we investigated the binding of divalent Ca^2+^ ions to heparin through titration of the sodium heparin sample with 0 to 100 mM CaCl_2_ and ^23^Na NMR measurements. After adding 5 mM Ca^2+^, a considerable reduction in ^23^Na signal linewidth was observed (Figure 2a). Through titration, a gradual change in the chemical shift and linewidth of the ^23^Na NMR signal towards those of the control sample was observed, indicating Ca^2+^ binding-induced release of sodium ions from heparin and exchange between the free and bound sodium ions occurring in the fast exchange regime with respect to NMR chemical shift timescale (*k*_ex_ >> Δω ≅ 96 s^−1^). Importantly, the Ca^2+^ concentration-dependent change in ^23^Na signal linewidth showed a limiting value of around 10.1 ± 0.1 Hz (Figure 2b), considerably larger than the value of 5.3 ± 0.1 Hz obtained for the control sample. The limiting linewidth value was almost fully achieved already at around 40–60 mM CaCl_2_ concentration and the further increase in CaCl_2_, concentration to 100 mM had little impact on ^23^Na signal linewidth. These data suggest the presence of at least two sodium-binding sites on heparin, one with a weak affinity that is readily substituted by added Ca^2+^ ions in the range up to ~ 50 mM concentration, the other(s) with a stronger affinity that remains occupied even at added Ca^2+^ concentrations as high as 100 mM.

**Figure 3:**
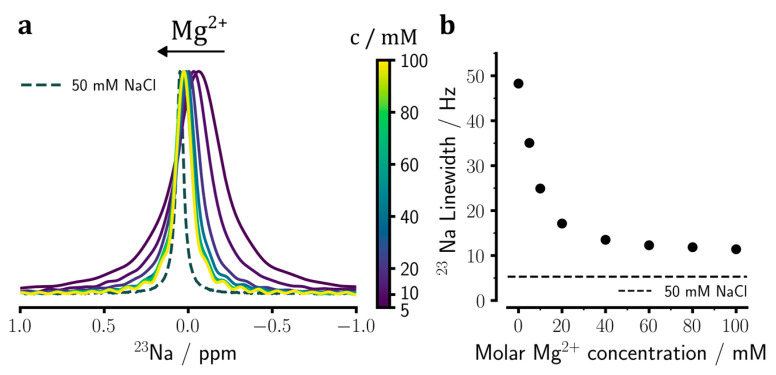
^23^Na NMR spectral changes of sodium heparin caused by the addition of Mg^2+^ ions and the consequent release of sodium ions from heparin. (**a**) Upon titration of sodium heparin with 0 to 100 mM MgCl_2_, the ^23^Na chemical shift gets narrower and moves downfield towards the chemical shift of the reference NaCl sample (see Figure 1). (**b**) Changes in the ^23^Na signal linewidth in dependence of added Mg^2+^ ion concentration, showing a monotonic decrease approaching a value slightly larger than the linewidth of the reference NaCl sample (represented by the dashed line). The spectra shown in (**a**) are re-scaled according to their peak intensities. The error bars in (**b**) are comparable in size to the symbols and, therefore, barely visible.

Subsequently, we studied the binding of divalent Mg^2+^ ions to heparin through similar ^23^Na NMR-based titration experiments with MgCl_2_ (Figure 3a). Titration with 0 to 100 mM Mg^2+^ ions led to changes in ^23^Na chemical shifts from −0.095 ppm to 0.026 ppm and in the linewidths from 48.3 Hz to 11.4 Hz. In line with Ca^2+^ ion titration data (Figure 2b), the Mg^2+^ concentration-dependent change in linewidth further supported the presence of more than one sodium-binding site on heparin (Figure 3b). Furthermore, the comparison between the limiting line widths (11.4 Hz for Mg^2+^ vs. 10.1 Hz for Ca^2+^) suggested that the affinity of Mg^2+^ ions for the weaker sodium ion-binding site on heparin was lower than that of Ca^2+^ ions. In the absence of titration data at much larger concentrations, however, the affinities of Ca^2+^ and Mg^2+^ ions for the stronger binding site(s) on heparin could not be compared.

### 2.2. Rotational Mobility of Released Sodium Ions from Heparin

To further investigate how the addition of Ca^2+^ or Mg^2+^ ions affects sodium ion binding to heparin, the longitudinal spin-lattice (*T*_1_) relaxation times of ^23^Na were measured in sodium heparin samples in the absence or presence of added ions through standard inversion-recovery experiments.

**Figure 4:**
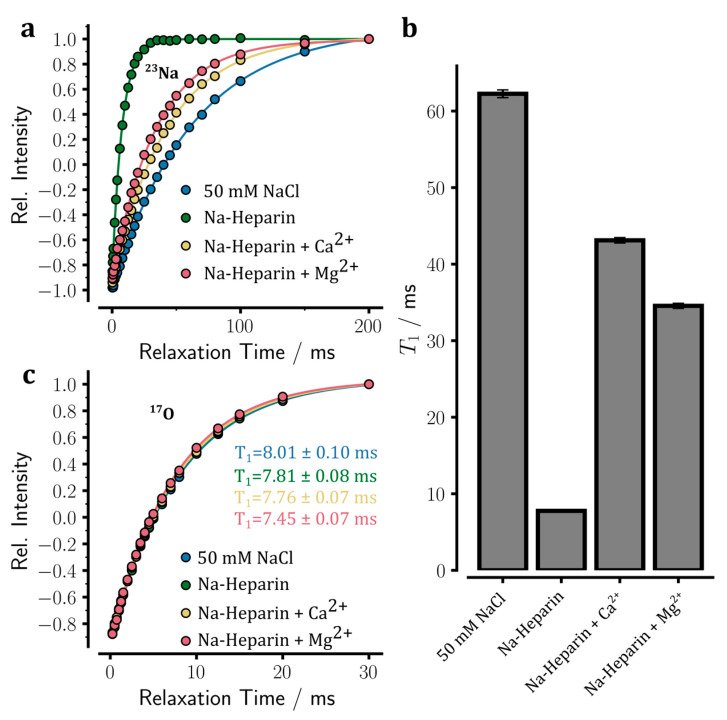
Longitudinal spin-lattice (*T*_1_) relaxation of ^23^Na NMR signals in sodium heparin samples determined through standard inversion-recovery NMR experiments. (**a**) The ^23^Na inversion-recovery data are shown as relative intensity versus relaxation time (duration of relaxation delay) for the following samples: sodium heparin sample without added ion, sodium heparin samples with 100 mM CaCl_2_ or MgCl_2_ added, and a reference NaCl solution. Upon the addition of Mg^2+^ and especially Ca^2+^ ions, the relaxation-induced recovery of ^23^Na signals becomes slower and approaches but does not reach, that of the reference NaCl sample. (**b**) The obtained ^23^Na *T*_1_ relaxation times of the measured samples. The ^23^Na *T*_1_ data show partial Ca^2+^ or Mg^2+^ ion-dependent release of sodium ions from heparin, which is stronger in the case of Ca^2+^ ions. (**c**) The ^17^O inversion-recovery data of the above samples show only slight differences in the viscosity-dependent ^17^O *T*_1_ of water.

In the control sample (50 mM NaCl), the intensity recovery obeyed a mono-exponential recovery curve consistent with Na^+^ ions in the fast extreme-narrowing regime (Figure 4a). Even in the sodium heparin sample in which the heparin-bound sodium ions are expected to have a much slower rotational correlation time than free sodium ions, the intensity recovery data did not show a clear deviation from the mono-exponential recovery curve either (Figure 4a). The ^23^Na *T*_1_ relaxation times obtained through fitting to a mono-exponential recovery function (see Equation (1) in Section 4) were 62.3 ± 0.5 ms in the control NaCl solution and 7.8 ± 0.0 ms in the sodium heparin sample (Figure 4b). Notably, the ^23^Na *T*_1_ relaxation time of 62.3 ms in the control NaCl sample was almost identical to the ^23^Na *T*_2_ of 60.2 ms estimated through the linewidth of the ^23^Na signal (see above, the corresponding relaxation rates were 16.1 and 16.6 s^−1^, respectively). The small difference of around 0.5 s^−1^ in relaxation rates are likely to be caused by *B*_0_ field inhomogeneity broadening and the inherent noise in the spectrometer frequency lock system. Unlike the control sample, however, a considerable difference was observed between the ^23^Na *T*_1_ relaxation time of 7.8 ms in the sodium heparin sample and the linewidth-based *T*_2_ of 6.6 ms: the corresponding relaxation rates were 128.2 and 151.7 s^−1^. The difference of around 23.5 s^−1^ in relaxation rates is too large to be entirely caused by the *B*_0_ field inhomogeneity and lock system noises. Instead, it suggests the possibility that the system is partially outside the extreme-narrowing regime. Indeed, the *T*_1_/*T*_2_ ratio of ~1.18 is consistent with a τ_c_ of ca. 0.88 ns. Under this condition where ω_0_τ_c_ is ~0.585, the difference in *T*_1_ or *T*_2_ relaxation times of the central and satellite transitions are too small to lead to a clear bi-exponential intensity recovery in *T*_1_ relaxation experiments or a double-Lorentzian signal line shape. Another possibility is that an exchange process between free and heparin-bound sodium ions occurring at tens of milliseconds timescale leads to exchange-mediated contribution to *T*_2_, but not *T*_1_, relaxation rates.

The almost eight-fold difference in ^23^Na *T*_1_ of these two samples potentially originated from two factors, (i) the slower rotational correlation time of heparin-bound sodium ions than free sodium ions, and (ii) the larger local electric field gradient present at the site of heparin-bound ^23^Na nuclei compared to free symmetrically-hydrated sodium ions. The possible contribution of viscosity changes to ^23^Na *T*_1_ can be excluded, as the viscosity-dependent ^17^O *T*_1_ times do not show any significant difference between these two samples (Figure 4c) [22]. If we assume a ^23^Na quadrupolar coupling constant (QCC, χ) of 500–2000 kHz, typical values reported for sodium carboxylate salts in literature [29,30], then the ^23^Na *T*_1_ of 7.8 ms in the sodium heparin sample would imply a τ_c_ of ~6–130 ps (longer τ_c_ values at smaller QCCs). Notably, the lower limit of 6 ps coincides with the reported τ_c_ of free sodium ions at the same temperature [31]. In comparison, the upper limit of 130 ps is too short of pushing the quadrupolar relaxation of sodium ions away from the extreme-narrowing regime. If we take τ_c_ of ca. 0.88 ns estimated above from the *T*_1_/*T*_2_ ratio, then a ^23^Na QCC of around 234 kHz would be needed to reproduce the (average) *T*_1_ and *T*_2_ values. These QCC values are unrealistically small for the sodium ions bound specifically to negatively charged carboxylate or sulfate groups of heparin [29,30]. On the other hand, they seem too large for sodium ions bound non-specifically to the negatively charged layer of the heparin surface. Instead, the estimated QCC of around 234 kHz could arise from a dynamic exchange of sodium ions between these two modes of binding to heparin.

Next, we investigated how the addition of Ca^2+^ or Mg^2+^ ions would affect the ^23^Na *T*_1_ relaxation in the sodium heparin sample. As shown in Figure 4b, the mono-exponetial pattern of intensity recovery was preserved upon the addition of 100 mM CaCl_2_ or MgCl_2_. However, the ^23^Na *T*_1_ rose from 7.8 ± 0.0 in the sodium heparin sample to 43.1 ± 0.4 and 34.6 ± 0.3 ms, respectively. Notably, the ^23^Na *T*_2_ relaxation rates estimated from linewidths were 25.7 and 30.9 s^−1^, respectively, after the addition of Ca^2+^ or Mg^2+^ ions, which were only slightly different from the corresponding *T*_1_ relaxation rates of 23.2 ± 0.2 and 28.9 ± 0.3 s^−1^. The ^23^Na *T*_1_ values obtained after the addition of Ca^2+^ or Mg^2+^ ions were smaller than the value of 62.3 ± 0.5 ms measured in the control NaCl sample, indicating that Ca^2+^ or Mg^2+^ ion-induced sodium release from heparin was incomplete. Assuming that the observed ^23^Na *T*_1_ relaxation rates were population-weighted averages of the relaxation rates of free and heparin-bound sodium ions, it was found that Ca^2+^ ions had released 93.6 ± 1.8% of the heparin-bound sodium ions, while the corresponding value for Mg^2+^ ions was 88.5 ± 1.8%. These results, therefore, support the higher strength of Ca^2+^ than Mg^2+^ ions in releasing sodium ions from heparin.

### 2.3. Translational Mobility of Released Sodium Ions from Heparin

**Figure 5:**
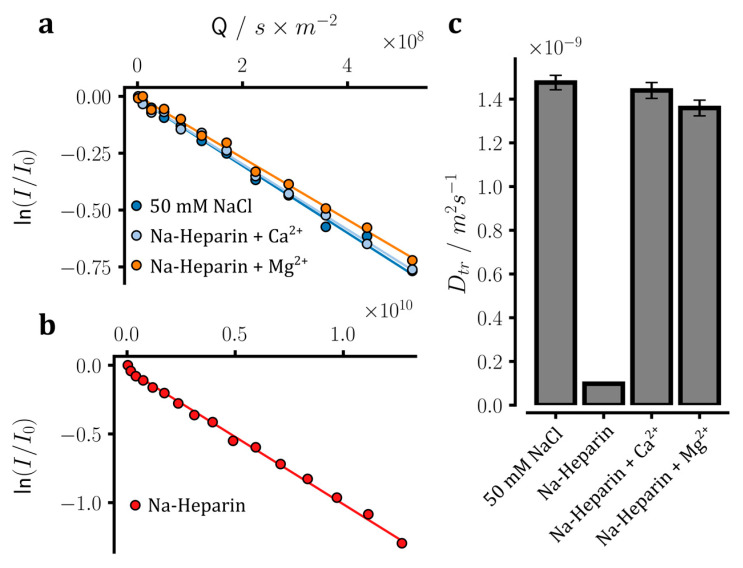
Translational diffusion of sodium ions measured in heparin samples through ^23^Na or ^1^H-based pulse field gradient NMR diffusion experiments. (**a**,**b**) Gradient-dependent decrease in the intensity of ^23^Na NMR signals in sodium heparin samples in the presence of 100 mM CaCl_2_ or MgCl_2_, as well as in the reference NaCl sample. Due to the fast relaxation of ^23^Na signals in the sodium heparin sample without added ions and the severe loss of ^23^Na signal intensity, no ^23^Na NMR diffusion experiment could be conducted for this sample. Instead, as shown in (**b**), the diffusion coefficient of sodium ions in the heparin-bound form was estimated through a ^1^H-based NMR diffusion experiment, assuming that the heparin molecules and sodium ions are rigidly bound and diffuse together. (**c**) The obtained translational diffusion coefficients (*D*_tr_) of sodium ions in the measured samples. The diffusion data are consistent with the partial release of sodium ions from heparin after the addition of Mg^2+^ and especially Ca^2+^ ions.

Subsequently, we investigated how the translational mobility of sodium ions is affected by the addition of Ca^2+^ or Mg^2+^ ions to sodium heparin. To this end, the diffusion coefficient of sodium ions was measured through the PFG-NMR diffusion method (Figure 5a,b). In these measurements, the *z*-axis gradient-dependent attenuation of NMR signal intensities during diffusion delays is interpreted as diffusive displacement of the underlying molecules/ions along the *z*-axis, and thereby diffusion coefficients of respective molecules/ions are determined [32]. The free sodium ions in the control NaCl sample showed a diffusion coefficient of 1.48 ± 0.03 × 10^−9^ m^2^·s^−1^, which is in good agreement with previous reports [18,19,31]. The heparin-bound sodium ions, however posed a challenge for NMR dffusion measurements as the relatively slow diffusion of the bound ions and the severe relaxation of the ^23^Na signal during the required tens of milliseconds-long diffusion delays would not leave any appreciable ^23^Na signal to be measured. Assuming that the heparin-bound sodium ions diffuse together with the heparin, it was possible to indirectly determine the diffusion coefficient of bound sodium ions through ^1^H-based PFG-NMR measurements (Figure 5b). In this way, the diffusion coefficient of heparin-bound sodium ions was determined at 9.80 ± 0.19 × 10^−11^ m^2^·s^−1^, which is almost 15 times smaller than the diffusion coefficient of free sodium ions. It is worth noting that the free and heparin-bound sodium ions showed a less marked difference in their ^23^Na *T*_1_ than their diffusion coefficients (eight-versus 15-fold, see above), which suggests that the sodium ions probably enjoy a considerable degree of local rotational mobility in the heparin-bound form.

Upon addition of 100 mM CaCl_2_ or MgCl_2_ to heparin, the diffusion coefficient of sodium ions increased to 1.44 ± 0.04 × 10^−9^ and 1.36 ± 0.04 × 10^−9^ m^2^·s^−1^, respectively. Assuming that the observed diffusion coefficients of sodium ions were population-weighted averages of the free and heparin-bound sodium ions, it was found that 97.1 ± 2.7% and 91.3 ± 2.9% of sodium ions were released by Ca^2+^ and Mg^2+^ ions, respectively. These results are in qualitative agreement with the above-mentioned ^23^Na *T*_1_ relaxation data and further support the higher strength of Ca^2+^ ions than Mg^2+^ ions in releasing sodium ions from heparin.

### 2.4. Rotational and Translational Mobility of Titrated Metal Ions, Case of ^27^Al Ions

The data presented above demonstrate the power of ^23^Na NMR-based competition assays in detecting and quantifying metal ion binding to heparin. To explore whether more direct NMR methods could be used to monitor metal ions binding to heparin, we turned our attention to the nuclei of binding metal ions. Unfortunately, the NMR-active calcium and magnesium isotopes like ^43^Ca and ^25^Mg are relatively rare in nature and have very low gyromagnetic ratios (γ), compromising the sensitivity of NMR experiments and demanding specific NMR probes suitable for low-γ nuclei. Therefore, we chose aluminum ions, of which the ^27^Al nucleus has a natural abundance of ca. 100% and a fairly large gyromagnetic ratio. The ^27^Al nucleus has a spin quantum number of 5/2; hence it is quadrupolar [24]. As a result, its relaxation rate is significantly contributed by the quadrupolar relaxation mechanism, which is highly sensitive to binding events and the resultant changes in the chemical environment.

**Figure 6:**
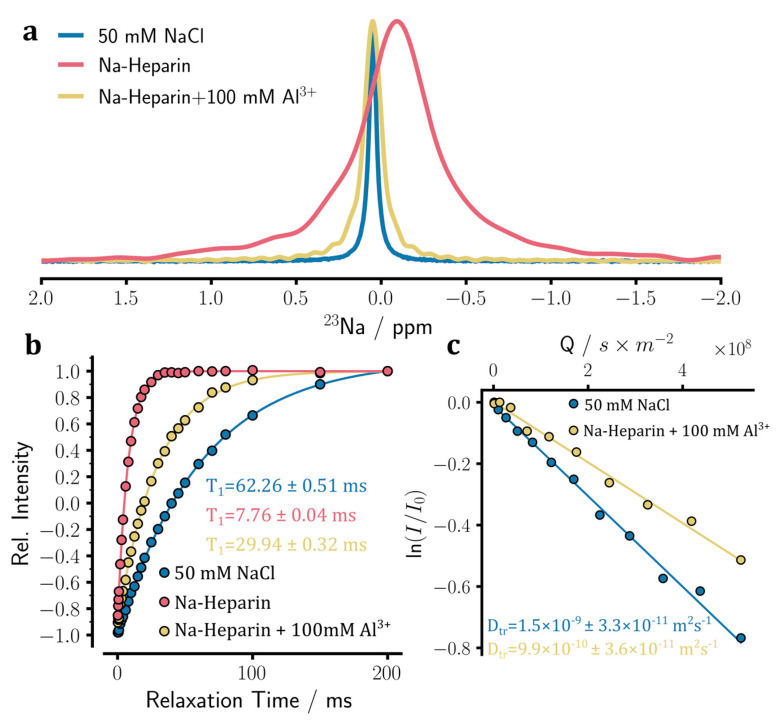
Aluminum ion binding to sodium heparin was monitored through ^23^Na NMR experiments. (**a**) 1D ^23^Na NMR spectra of sodium heparin, before and after the addition of 100 mM Al_2_(SO4)_3_. The spectrum of the reference NaCl solution is also shown. The addition of Al^3+^ ion leads to ^23^Na signal narrowing and peak displacement towards the chemical shift of free sodium ions. (**b**) Longitudinal spin-lattice (*T*_1_) relaxation of ^23^Na signals measured through standard inversion-recovery experiments. The *T*_1_ relaxation data indicate the partial release of sodium ions from heparin caused by the addition of Al^3+^ ions. (**c**) Translational diffusion coefficient (*D*_tr_) of sodium ions measured through pulse field gradient NMR diffusion experiments. The *D*_tr_ of sodium ions in the heparin with Al_2_(SO_4_)_3_ sample is larger than that of the heparin sample without added ions (see Figure 5c) but smaller than that of the reference NaCl sample. This is consistent with the partial release of sodium ions from heparin caused by added Al^3+^ ions.

The addition of 100 mM aluminum sulfate resulted in the release of sodium ions from heparin, as reflected in changes in the 1D ^23^Na NMR spectra and an increase in ^23^Na *T*_1_ and diffusion coefficients (Figure 6a–c). The Al^3+^ ion-induced increase of ^23^Na *T*_1_ to 29.9 ± 0.3 ms indicates the release of ca. 85% of sodium ions from heparin, which is considerably smaller than the corresponding values for Ca^2+^ (ca. 94%) and Mg^2+^ (ca. 89%). The relatively small increase of the ^23^Na diffusion coefficient to 9.9 ± 0.4 × 10^−10^ m^2^·s^−1^ suggested an even lower amount of sodium ion release (ca. 65%). In qualitative agreement with previous reports [11], the ^23^Na *T*_1_ times and diffusion coefficients indicated that the trivalent Al^3+^ ions were less strong than the divalent Ca^2+^ and Mg^2+^ ions in releasing sodium ions from heparin (the strength order was Ca^2+^ > Mg^2+^ > Al^3+^). It is, however, notable that the Al^3+^ ion-induced release of sodium ions from heparin indicates a larger affinity of Al^3+^ than sodium ions for the respective binding site on heparins, which is not consistent with the order of affinities reported in ref. [11] based on atomic absorption experiments.

**Figure 7:**
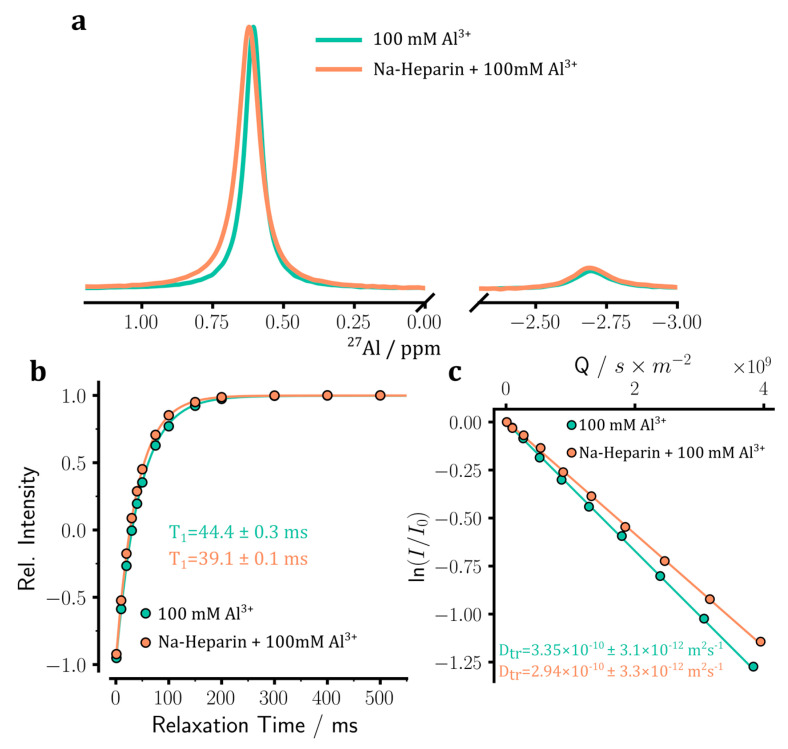
Aluminum ion binding to sodium heparin was monitored through ^27^Al NMR experiments. (**a**) 1D ^27^Al NMR spectra of 100 mM Al_2_(SO_4_)_3_ sample, in the absence or presence of sodium heparin. The major and minor peaks, respectively, corresponding to [Al(H_2_O)_6_]^3+^ and [Al(H_2_O)_5_SO_4_]^+^ species, are shown. The addition of sodium heparin leads to slight displacement and broadening of the ^27^Al signal. (**b**) Longitudinal spin-lattice (*T*_1_) relaxation of ^27^Al signals measured through standard inversion-recovery experiments. (**c**) Translational diffusion coefficient (*D*_tr_) of Al^3+^ ions measured through pulse field gradient NMR diffusion experiments. Upon the addition of heparin, a small decrease in ^27^Al *T*_1_ relaxation time (**b**) and *D*_tr_ coefficient (**c**) is observed, indicating the partial binding of Al^3+^ ions to heparin.

Finally, we measured the 1D ^27^Al NMR spectra of a 100 mM aluminum sulfate solution in the absence or presence of sodium heparin (5 IU/mL). As shown in Figure 7a, the 1D ^27^Al NMR spectrum of aluminum sulfate showed two peaks, a major peak (88.6 ± 0.2%) centered at around 0.9 ppm and a minor peak (11.4 ± 0.2%) around −2.4 ppm. A previous study has shown that the major peak corresponds to [Al(H_2_O)_6_]^3+^ species, while the minor peak has its origin in [Al(H_2_O)_5_SO_4_]^+^ species [33]. Upon the addition of heparin, the chemical shifts and linewidths of the two ^27^Al signals showed changes suggesting the binding of aluminum ion species to heparin. Further direct evidence for the binding of Al^3+^ ions to heparin was provided by ^27^Al *T*_1_ relaxation measurements. Upon addition of heparin, the ^27^Al *T*_1_ relaxation times decreased from 44.4 ± 0.3 ms to 39.1 ± 0.1 ms for the major peak (Figure 7b) and from 32.4 ± 0.1 ms to 30.5 ± 0.1 ms for the minor peak. The translational diffusion coefficient of Al^3+^ ions changed from 3.4 ± 0.03 × 10^−10^ m^2^·s^−1^ in the control sample to 2.9 ± 0.03 × 10^−10^ m^2^·s^−1^ in the heparin sample (Figure 7c). Assuming that the bound Al^3+^ ions diffuse together with heparin and have a diffusion coefficient identical to that of heparin, it is estimated that around 83% of Al^3+^ ions are in the free and only 17% are in the bound states. The ^23^Na and ^27^Al NMR provided complementary probes of binding/un-binding these metal ions to/from heparin.

## 3. Discussion

Our results showed that metal ion binding to heparin could be monitored through a ^23^Na NMR-based competition assay. The addition of Ca^2+^ (Figure 2), Mg^2+^ (Figure 3), and Al^3+^ (Figure 6) ions to sodium heparin led to the release of sodium ions from heparin, as revealed by metal ion-induced changes in ^23^Na chemical shifts and linewidths (Figure 2, Figure 3 and Figure 6), *T*_1_ relaxation times (Figure 4 and Figure 6) and diffusion coefficients (Figure 5 and Figure 6). Analysis of ^23^Na NMR data demonstrated the presence of more than one metal ion-binding site on heparin and supported the affinity order of Ca^2+^ > Mg^2+^ > Al^3+^ for the site with lower affinity. In the case of Al^3+^, complementary ^27^Al NMR experiments provided direct evidence for Al^3+^ ions binding to heparin (Figure 7).

Heparins have a high negative charge density because of carboxylate and particularly sulfate groups in their structure (see Scheme 1) [1]. It is therefore expected that heparins act as polyelectrolytes and in dilute solutions covered by a layer of positive counterions, as predicted by Manning’s counterion condensation model [34] or Poisson-Boltzmann theory [35]. In addition to this rather non-specific mode of counterion binding to heparin, the positively charged metal ions can be more specifically engaged in binding with negatively charged groups in heparin, where specific interactions beyond electrostatic interaction stabilize the ion-heparin binding. The ^23^Na NMR data reported here confirm the presence of more than one sodium ion binding site on heparin, in which the site with weaker binding affinity is substituted by added divalent Ca^2+^ and Mg^2+^ and trivalent Al^3+^ ions. Our data do not allow identifying the sodium binding sites on heparin. Nevertheless, the lower melting point and higher solubility of sodium acetate than sodium sulfate suggest that the lattice energy of sodium acetate is lower than sodium sulfate. Accordingly, it appears reasonable to speculate that the carboxylate groups may form the weak binding site, while the sulfate groups are more likely involved in the strong binding mode. The ^23^Na NMR-based competition data support the relative affinity order of Ca^2+^ > Mg^2+^ > Al^3+^ > Na^+^ ions for the weak binding site on heparin. Regarding the strong binding site, the data presented here does not provide a relative affinity order. It is, however, interesting to note that the addition of 100 mM Ca^2+^, Mg^2+^, or Al^3+^ ions did not displace the residual bound sodium ions at concentrations as low as 7 mM or lower (<15% of total Na^+^ concentration of mM, see above). As a result, the Na^+^ ion seems to have a larger affinity than the studied divalent and trivalent ions for the strong binding site on heparin. The suggested affinity orders for the weak and strong binding sites of heparin are partially consistent with a previous atomic absorption-based report [11], potentially because of its different sensitivity to various modes of ion-heparin binding. Our data highlights the importance of site-specific affinity data in ion-heparin binding studies, especially when contradictory results are obtained through various techniques.

In accordance with partial deviation from the extreme-narrowing regime, the ^23^Na *T*_1_ and *T*_2_ relaxation times of the sodium heparin sample were different from each other. The ^23^Na *T*_1_ and *T*_2_ relaxation rates could be explained by a τ_c_ of ~0.88 ns and a ^23^Na quadrupolar coupling constant of ~ 234 kHz. The latter value lies between the typical quadrupolar coupling constants of sodium carboxylate ions [29,30] and the values expected for sodium counterions non-specifically condensed on heparin undergoing dynamic exchange with the surrounding ionic atmosphere and bulk solution. Accordingly, we hypothesize that a sodium ion exchange process between the specific and non-specific binding sites of heparin partially averages the relaxation-active quadrupolar coupling constants and leads to an effective intermediate coupling constant. A complete analysis of this complex exchange process involves several unknown parameters and demands extensive relaxation rate measurements beyond the scope of the current study.

The ubiquitous presence of negatively charged proteoglycans in the extracellular space can act as buffers for positively charged metal and organic ions and thereby influence various physiological and pathophysiological processes outside the cells [12,13]. In addition, it is known that ions such as Ca^2+^ or Mg^2+^ can alter the conformation of heparin and consequently modulate their functions, e.g., in host defense against pathogens [10]. In neurodegenerative diseases, heparins are known to trigger pathogenic aggregation of proteins such as tau protein in a metal ion-dependent manner on the one hand [7,36,37,38] and act as neuroprotective agents on the other hand [9]. Considering various modes of ions binding to heparin, as shown here, it is interesting to explore the range of structural and activity changes in heparin in dependence on ion types and binding sites.

In summary, we present a multinuclear quadrupolar NMR study of metal ions binding to heparins. Using a ^23^Na NMR-based competition assay involving ^23^Na NMR relaxation and diffusion measurements, we detect and quantify the binding of metal ions Ca^2+^, Mg^2+^, and Al^3+^ to sodium heparin, support the presence of more than one ion binding site on heparin undergoing dynamic exchange and provide a relative affinity order for the studied ions. Combined with other methods of studying ion-heparin binding, the presented method can provide a more detailed picture of ion-heparin binding and its potential structural and functional consequences.

## 4. Materials and Methods

Magnesium chloride, MgCl_2_, calcium chloride, CaCl_2_, and aluminum sulfate, Al_2_(SO_4_)_3_, were purchased from Sigma. The unfractionated heparin (high-molecular-weight, ~10–20 kDa) at a concentration of 50,000 IU/mL was obtained from Ratiopharm (Ulm, Germany).

NMR experiments were conducted at a Bruker spectrometer with a proton Larmor frequency of 400.13 MHz equipped with a room-temperature triple resonance broadband (TBO) probe. Unless stated otherwise, the NMR samples contained 5 IU/mL sodium heparin dissolved at 99.95% D_2_O, to which MgCl_2_, CaCl_2_, or Al_2_(SO_4_)_3_ at specified concentrations were added. The deuteron signal was used for spectrometer frequency locking and chemical shift referencing (4.700 ppm). For natural abundance ^23^Na, ^27^Al, and ^17^O NMR experiments, the inner broadband coil of the TBO probe was tuned and matched at ~ 105.84, 104.26, and 54.24 MHz, respectively, corresponding to the Larmor frequencies of ^23^Na, ^27^Al, and ^17^O nuclei. NMR measurements were performed at 298 K, controlled to ±0.05 K by a Bruker VT unit calibrated using the residual proton signals of a standard deuterated methanol sample.

The longitudinal spin-lattice (*T*_1_) relaxation of ^23^Na, ^27^Al, and ^17^O was studied through standard inversion-recovery experiments. In ^23^Na *T*_1_ experiments, 26 relaxation delays (*t*) of 0.25, 0.5, 0.75, 1, 2, 3, 4, 6, 8, 10, 12.5, 15, 17.5, 20, 25, 30, 35, 40, 45, 50, 60, 70, 80, 100, 150 and 200 ms were used. The 16 relaxation delays used for ^27^Al *T*_1_ experiments were 1, 10, 20, 30, 40, 50, 75, 100, 150, 200, 300, 400, 500, 750, 1000, 2000 ms. The corresponding values for ^17^O *T*_1_ experiments were 0.25, 0.5, 0.75, 1, 1.25, 1.5, 2, 2.5, 3, 3.5, 4, 4.5, 5, 6, 7, 8, 10, 12.5, 15, 20, 30 ms. In all *T*_1_ experiments, sufficiently long total recycle delays (*d*_1_ + acq.) were used in order to ensure complete Boltzmann magnetization of the studied nuclei. Assuming a fast “extreme narrowing” regime in which relaxation-dependent recovery of signal intensities of these quadrupolar nuclei obeys a single-exponential recovery equation (see below), the intensity (*I*) versus relaxation delay (*t*) data were fitted to:

(1) $$I=a\;\left(1-2be^{-t/T_{1}}\right)$$

through which the *T*_1_ relaxation times were obtained.

The (translational) diffusion coefficient (*D*) of sodium and aluminum ions was measured through the ^23^Na- or ^27^Al-based pulse-field-gradient (PFG) NMR diffusion method using the standard stebpgp1s pulse sequence. The known diffusion coefficient of residual HDO in 99.8% D_2_O at 25 °C (1.900 × 10^−9^ m^2^·s^−1^) was used for gradient calibration. The magnetic field z-gradient-based intensity attenuation data were fitted to the standard Stejskal-Tanner (ST) equation,

(2) $$I=I_{0}\;e^{-DQ}$$

with Q=γ2δ2(Δ−δ3)g2, where *γ* is the gyromagnetic ratio of the corresponding nucleus, g is the gradient strength and Δ and δ are the big and little diffusion delays, respectively. The big and little diffusion delays were 20 and 5 ms for ^23^Na and 75 and 7 ms for ^27^Al NMR diffusion experiments, respectively. In the ^1^H-based PFG-NMR experiment used to determine the diffusion coefficient of heparin-bound ions, the big and little diffusion delays were 50 and 5 ms, respectively.

The ^23^Na, ^27^Al, and ^17^O nuclei have spins *I* = 3/2, 5/2, and 5/2, respectively. Nuclei with spins I>12 have non-zero electric quadrupole moment (*eQ*), and its anisotropic interaction with the local electric field gradient (EFG) leads to an additional NMR relaxation mechanism called quadrupolar relaxation. In general, the quadrupolar relaxation of half-integer spin contains I+12 components, of which one belongs to the single central transition (CT: |−1/2〉⟷|+1/2〉) and I−12 components belong to the corresponding pairs of satellite transitions (ST: | ± 1/2〉⟷| ± 3/2〉 for spin-3/2 ^23^Na and | ± 1/2〉⟷| ± 3/2〉 and | ± 3/2〉⟷| ± 5/2〉 for spin-5/2 ^27^Al and ^17^O). However, within the so-called fast “extreme-narrowing” regime where $\omega_{0}\tau_{c}\;\ll 1$ (ω_0_, Larmor frequency, τ_c_, rotational correlation time associated with the stochastic reorientation of the EFG tensor), the relaxation rates of the central and satellite transitions become identical and, therefore a single-exponential relaxation pattern is recovered. Under this regime, the *T*_1_ and *T*_2_ relaxation times are identical and inversely proportional to τ_c_, according to

(3) $$\frac{1}{T_{1}}=\frac{1}{T_{2}}=\frac{3\pi^{2}}{10}\left(\frac{2I+3}{I^{2}\left(2I-1\right)}\right)\chi^{2}\left(1+\frac{\eta^{2}}{3}\right)\;\tau_{c}$$

where *I* is the spin quantum number, $\chi =e^{2}Qq_{zz}/h$ is the quadrupolar coupling constant (QCC, C_Q_), *Q* is the quadrupole moment, and η is the asymmetry parameter representing the deviation of the electric field gradient (EFG) tensor *eq* from axial symmetry. When τ_c_ and/or ω_0_ increase and the system enters the so-called “slow regime” where $\omega_{0}\tau_{c}\;\sim \;1$ or larger, the *T*_1_ and *T*_2_ times corresponding to the CT and ST components become distinct. In the case of spin-3/2 nuclei such as ^23^Na, the two relaxation times are given by Equations (4)–(7) [17,23,25,26]:

(4) $$\frac{1}{T_{1,\;CT}}=\frac{3\pi^{2}}{10}\left(\frac{2I+3}{I^{2}\left(2I-1\right)}\right)\chi^{2}\left(1+\frac{\eta^{2}}{3}\right)\left(\frac{\tau_{c}}{1+4\omega_{0}^{2}\tau_{c}^{2}}\right)$$

(5) $$\frac{1}{T_{1,\;ST}}=\frac{3\pi^{2}}{10}\left(\frac{2I+3}{I^{2}\left(2I-1\right)}\right)\chi^{2}\left(1+\frac{\eta^{2}}{3}\right)\;\left(\frac{\tau_{c}}{1+\omega_{0}^{2}\tau_{c}^{2}}\right)$$

(6) $$\frac{1}{T_{2,CT}}=\frac{3\pi^{2}}{20}\left(\frac{2I+3}{I^{2}\left(2I-1\right)}\right)\chi^{2}\left(1+\frac{\eta^{2}}{3}\right)\left(\frac{\tau_{c}}{1+\omega_{0}^{2}\tau_{c}^{2}}+\frac{\tau_{c}}{1+4\omega_{0}^{2}\tau_{c}^{2}}\right)$$

(7) $$\frac{1}{T_{2,ST}}=\frac{3\pi^{2}}{20}\left(\frac{2I+3}{I^{2}\left(2I-1\right)}\right)\chi^{2}\left(1+\frac{\eta^{2}}{3}\right)\left(\tau_{c}+\frac{\tau_{c}}{1+\omega_{0}^{2}\tau_{c}^{2}}\right)$$

The bi-exponential longitudinal spin-lattice and transverse spin relaxation of ^23^Na nuclei can be expressed as:

(8) Mz(t)−M0=M0 (0.8etT1,CT+0.2etT1,ST)

(9) Mx,y(t)=M0 (0.4etT2,CT+0.6etT2,ST)

in which *M*_z_ and *M*_x,y_ are longitudinal and transverse magnetizations and *M*_0_ is the thermal equilibrium (Boltzmann) magnetization. For the calculation of ^23^Na relaxation rates in dependence of *τ_c_* or *χ* on the basis of Equations (3)–(7), an asymmetry parameter η of 0.4 was assumed. With further increase in *τ_c_* when $\omega_{0}\tau_{c}>{\left(\frac{\omega_{0}}{\omega_{Q}}\right)}^{2}$ (*ω_Q_* is quadrupolar coupling constant *C_Q_* in angular frequency units), the systems enter the so-called “ultra-slow” regime in which the spin relaxation deviates from Redfield’s relaxation theory [26,28]. The “ultra-slow” regime is not relevant in our study and therefore is not described further.

## Data Availability

The data of this study can be shared upon reasonable request from the corresponding author.
